# Autoantibodies predict type 1 diabetes after gestational diabetes – a 23-year cohort study

**DOI:** 10.3389/fendo.2023.1286375

**Published:** 2023-12-20

**Authors:** Kaisu Luiro, Anna-Maaria Auvinen, Juha Auvinen, Jari Jokelainen, Ilkka Järvelä, Mikael Knip, Juha S. Tapanainen

**Affiliations:** ^1^Department of Obstetrics and Gynecology, University of Helsinki and Helsinki University Hospital, Helsinki, Finland; ^2^Research Unit of Clinical Medicine, University of Oulu, Oulu, Finland; ^3^Medical Research Center, Oulu University Hospital and University of Oulu, Oulu, Finland; ^4^Research Unit of Population Health, University of Oulu, Oulu, Finland; ^5^Northern Finland Birth Cohorts, Infrastructure for Population Studies, Faculty of Medicine, University of Oulu, Oulu, Finland; ^6^Department of Obstetrics and Gynecology, Kuopio University Hospital, Kuopio, Finland; ^7^Pediatric Research Center, New Children’s Hospital, University of Helsinki and Helsinki University Hospital, Helsinki, Finland; ^8^Research Program for Clinical and Molecular Metabolism, Faculty of Medicine, University of Helsinki, Helsinki, Finland; ^9^Tampere Center for Child Health Research, Tampere University Hospital, Tampere, Finland; ^10^Department of Obstetrics and Gynecology, HFR – Cantonal Hospital of Fribourg and University of Fribourg, Fribourg, Switzerland

**Keywords:** autoantibody, GDM, insulin, ICA, OGTT, prediction, type 1 diabetes, type 2 diabetes

## Abstract

**Objective:**

To study the predictive value of autoantibodies for type 1 (T1DM) and type 2 (T2DM) diabetes morbidity after gestational diabetes (GDM) in a 23-year follow-up study.

**Design:**

Prospective population-based cohort study.

**Methods:**

We studied 391 women with GDM, and 391 age- and parity-matched controls, who delivered in 1984–1994. Four autoantibodies were analysed in first-trimester blood samples: islet cell autoantibodies (ICAs), glutamic acid decarboxylase autoantibodies (GADAs), insulin autoantibodies (IAAs) and insulinoma-associated antigen-2 autoantibodies (IA-2As). Two follow-up questionnaires (1995–1996, 2012–2013) were sent to assess development of T1DM and T2DM. Predictive value of autoantibodies and clinical factors were analysed by conditional linear regression and ROC analyses.

**Results:**

Single autoantibody positivity was detected in 12% (41/342) of the GDM cohort and in 2.3% (8/353) of the control cohort. In the GDM cohort, 2.6% (9/342) tested positive for two autoantibodies and 2.3% (8/342) for three autoantibodies, whereas only one subject in the control cohort had two autoantibodies. ICA positivity was found in 12.5% of the cases, followed by GADA (6.0%), IA-2A (4.9%) and IAA (1.2%). In the control cohort, GADA positivity was found in 1.4%, IA-2A in 0.8%, IAA in 0.6%, and ICA in 0.3% of the subjects. Detection of ICA, GADA and/or IA-2A autoantibodies decreased T1DM-free survival time and time to diagnosis. All subjects with three positive autoantibodies developed T1DM within seven years from the GDM pregnancy. Development of T2DM after GDM occurred independent of autoantibody positivity.

**Conclusion:**

Development of T1DM can be reliably predicted with GADA and ICA autoantibodies during early pregnancy.

## 1 Introduction

Insulin sensitivity decreases during pregnancy along with increasing weight, adiposity and placental hormones, inducing insulin resistance to favour foetal growth. Gestational diabetes mellitus (GDM) develops when compensatory hyperinsulinaemia, that normally maintains an euglycemic state during pregnancy, can no longer counteract the increasing insulin resistance, and blood glucose levels rise (1). The prevalence of GDM is increasing worldwide and varies between 2 and 17% depending on the diagnostic criteria and genetic background of the studied population (2). The affected women are at high risk of developing type 2 diabetes (T2DM), and also type 1 (T1DM), later in life. Autoantibody positivity is a known risk factor for progression to T1DM (3), and autoantibodies predicting T1DM have been detected variably in 1-35% of women with GDM (4). However, long, prospective controlled studies aimed at assessing their role in the prediction of morbidity in both T1DM and T2DM after GDM, are lacking (5–25).

We have previously reported a prospective, 6-year cohort study of women with GDM and healthy controls, showing that positivity for islet cell autoantibodies (ICAs) and glutamic acid decarboxylase autoantibodies (GADAs), as well as GDM below the age of 30 years and the need for insulin treatment during pregnancy are associated with a high risk of subsequent progression to T1DM (15). Recently, we reported that during a 23-year follow-up of the same cohort, 5.7% of them developed T1DM and they were all diagnosed within 7 years after the GDM pregnancy, and their disease progression was predictable with high oral glucose tolerance test (OGTT) 2-h glucose levels, and associated with insulin treatment for GDM (16). Moreover, type 2 diabetes (T2DM) was diagnosed in 50.4% of the women with GDM, and the incidence remained linear until the end of the follow-up period.

Here we report the analysis of four autoantibodies; ICAs, GADAs, insulin autoantibodies (IAAs) and insulinoma-associated antigen 2 autoantibodies (IA-2As), evaluated during the first trimester of pregnancy from women with GDM and healthy controls in relation to the progression of T1DM and T2DM during a 23-year follow-up. Combined with the demographic and clinical data, we calculated the cumulative risk of one or more positive autoantibodies in disease progression and developed prediction models to assess the significance of independent clinical risk factors.

## 2 Methods

### 2.1 Study population

The study population has been previously described (16). This cohort study included 435 women with a singleton pregnancy and GDM, who delivered in the Oulu University Hospital, Finland, in 1984–1994. The control cohort of 435 women was pair-matched by age (± 2 years), parity (nulliparous, 1–3, or more than three deliveries) and date of delivery (± 2 days). All women were white. GDM was diagnosed by OGTT (n=363) or by insulin treatment (n=28). Subjects with a diagnosis based on multiple glucose measurements, or on abnormal HbA1c values, were excluded (n=44), and subsequently, 391 women with GDM, and 391 matched controls were included in the analyses.

Indications for OGTT included glucosuria, BMI ≥25 kg/m^2^, previous delivery of a macrosomic infant (≥4500 g) or expected macrosomic infant in the current pregnancy. A standard 2-h OGTT (75 g glucose load in 250 mL water) was performed after a 12-h overnight fasting. Three capillary whole blood samples were drawn: at baseline, at 60 min and 120 min. The cut-off values for the glucose concentrations were set according to the recommendation of the Finnish Diabetes Association: fasting, ≥4.8 mmol/L; 1-hour, ≥10.0 mmol/L; and 2-hour, ≥8.7 mmol/L. The blood samples were analysed using the HemoCue^®^ System (AB Leo Diagnostics, Helsingborg, Sweden) (1). The inter-assay coefficient of variation of the method was 3.8-4.0% at glucose concentration of 4.5-17.6 mmol/l. Any single abnormal value in the OGTT was considered diagnostic.

All women diagnosed with GDM were given nutritional advice. Insulin treatment was initiated, if at least two glucose values (fasting or preprandial) were ≥5.5 mmol/l or when one fasting or preprandial value was ≥5.5 mmol/l and one postprandial value was ≥7.8 mmol/l 1.5 hours after a meal in a 24-hour glucose profile.

All study participants signed an informed consent form. The Ethics Committee of the Northern Ostrobothnia Hospital District approved the study protocol.

### 2.2 Autoantibody analyses

A serum sample was taken during the first trimester of pregnancy for routine rubella screening. Diabetes-associated autoantibodies were analysed using a standard immunofluorescence method (ICA) or specific radiobinding assays (IAA, GADA and IA-2A) as previously described (18). All four autoantibodies were analysed successfully in 342 cases and 353 controls. ICA was analysed successfully in 352 cases and 354 controls, GADA in 350 cases and 354 controls, IA-2A in 344 cases and 353 controls, and IAA in 340 cases and 353 controls.

The cut-off level for ICA positivity was set at 2.5 Juvenile Diabetes Foundation units (JDFU) and for IAA, GADA, and IA-2A the cut-off levels were based on the 99^th^ percentile in nondiabetic Finnish subjects (N=105, 772 and 374, respectively). The cut-off limit for IAA positivity was set at specific binding of 54 nU/ml, for GADA 6.5 relative units (RU) and for IA-2A 0.43 RU. The disease sensitivity of the assays for ICA, IAA, GADA, and IA-2A were 100%, 78%, 79%, and 62%, respectively. The corresponding disease specificity was 98%, 100%, 97%, and 97%, respectively. All samples with IAA, GADA, or IA-2A levels between the 97^th^ and 99.5^th^ percentiles were reanalysed to confirm their status.

### 2.3 Questionnaire-based follow-up

Two questionnaires were sent to the study participants, first an invitation to participate in this study in 1995–1996 (1–11 years after pregnancy) with the first follow-up questionnaire and an informed consent form. Second follow-up questionnaire was sent in 2012–2013. 297 women with GDM and 297 control subjects (76%) took part in the study. Thirteen women in the GDM cohort (3.3%) and six women in the control cohort (1.5%) had died. The mean post-delivery follow-up time was 23.1 (range 18.7-28.8) years in the GDM cohort and 23.3 (range 18.9-30.1) years for the control cohort.

The questionnaires included questions about GDM treatment (diet or insulin), pre-pregnancy weight and height, progression to clinical diabetes, the type of diabetes, the time of diagnosis and diabetes medication.

### 2.4 Statistical analysis

Baseline demographic characteristics were analysed by one-way ANOVA. Development of T1DM and T2DM after pregnancy was assessed by Kaplan–Meier survival curves regarding (1) individual autoantibody positivity, and (2) number of positive autoantibodies. The time between blood sampling (taken in the first trimester) to the diagnosis of diabetes or to the end of follow-up was used as survival time (time-to-event). Subjects who did not answer the second questionnaire or who had died were censored at the end of their follow-up time or at the time of death. To evaluate the independent associations of each risk factor and to find the best predictive model for disease progression to diabetes, conditional logistic regression analysis and receiver operating characteristic (ROC) curves were constructed. AUC was used in the classification analysis. In model 1, the number of positive autoantibodies (0, 1, 2, or 3–4), age at the time of pregnancy (≤ 30 years vs. > 30), and non-insulin vs. insulin treatment for GDM were included as contributing factors. In model 2, positivity vs. negativity for each autoantibody, age at the time of pregnancy (≤ 30 years vs. > 30), and non-insulin vs. insulin treatment for GDM were included as contributing factors. The analyses were performed with IBM SPSS Statistics for Windows (versions 21 and 25, IBM, Armonk, NY) and RStudio (Boston, MA) software. The figures were produced using the ggplot2 (R package version 0.4.6., https://CRAN.R-project.org/package=survminer) and Adobe Illustrator (Adobe Systems, San Jose, CA).

## 3 Results

The demographic characteristics of the study population have been previously described (16). In brief, mean body weight and mean BMI ( ± SD) were higher in the GDM group than in the control group at the first trimester (69.5 ± 14.5 kg vs 61.7 ± 10.4 kg, *P*<0.001; 26.3 ± 5.2 kg/m^2^ vs 22.8 ± 3.5 kg/m^2^, *P*<0.001), as expected. Within the GDM cohort, women who later reported T1DM had lower first trimester mean BMI compared to those who later reported T2DM (24.2 ± 3.4 kg/m^2^ vs. 27.9 ± 5.7 kg/m^2^; *P*<0.001). The mean age of the GDM cohort at the time of second follow-up ( ± SD) was 54.7 ( ± 6.4) years, and that of the control cohort was 55.3 ( ± 6.4) years.

### 3.1 Autoantibody analyses

At least one autoantibody was found positive in 12% (41/342) of the GDM cohort and in 2.3% (8/353) of the controls (**Table 1**). Only one control subject (0.3%) had two positive autoantibodies, whereas in the GDM cohort, 2.6% (9/342) tested positive for two autoantibodies and 2.3% (8/342) for three autoantibodies. ICA positivity was found in 12.5% of the GDM cohort, followed by GADA (6.0%), IA-2A (4.9%) and IAA (1.2%). In the control cohort, GADA positivity was found in 1.4% of the subjects, IA-2A in 0.8%, IAA in 0.6%, and ICA in 0.3% of the subjects.

**Table 1:** Prevalence of the autoantibodies in the GDM and control cohort.

	Cases N=391*	Controls N=391*
	**% (N)**	**% (N)**
Positivity of autoantibodies^*^		
ICA	12.5 (44)	0.3 (1)
GADA	6.0 (21)	1.4 (5)
IA-2A	4.9 (17)	0.8 (3)
IAA	1.2 (4)	0.6 (2)
No. of positive autoantibodies^†^		
0	83.0 (284)	97.5 (344)
1	12.0 (41)	2.3 (8)
2	2.6 (9)	0.3 (1)
3	2.3 (8)	0 (0)
4	0 (0)	0 (0)

^*^ICA was analysed successfully from 352 cases and 354 controls, GADA from 350 cases and 354 controls, IA-2A 344 cases and 353 controls and IAA 340 cases and 353 controls.

^†^All four autoantibodies were analysed successfully from 342 cases and 353 controls.

**Figure 1:**
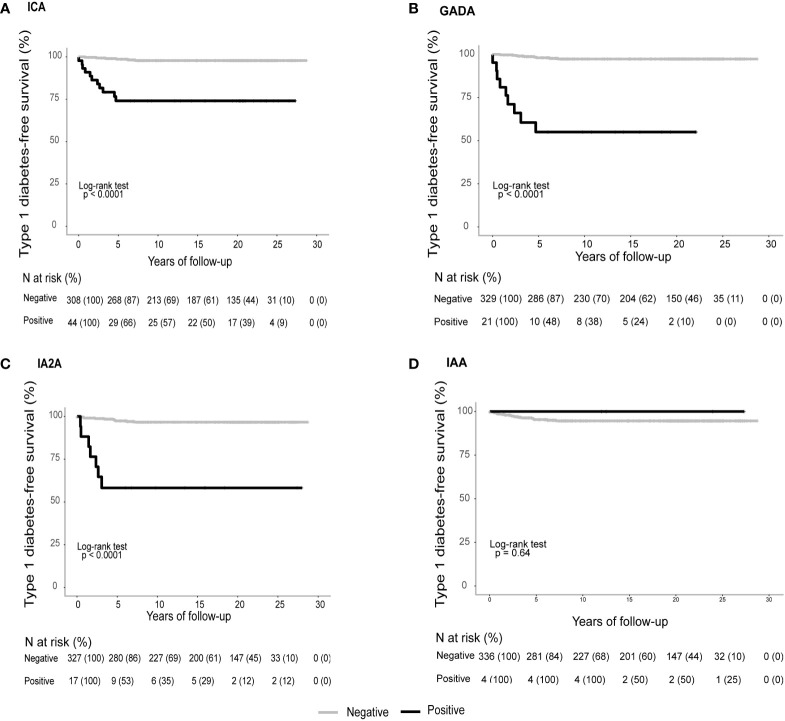
Mean (95% CI) T1DM -free survival time of women with vs without autoantibody positivity for **(A)** ICA 21.8 (18.3-25.4) vs 28.2 (27.8-28.7) years; **(B)** GADA 16.6 (10.7-22.6) vs 28.1 (27.7-28.6) years; **(C)** IA-2A 17.5 (11.1-23.9.) vs 27.9 (27.4-28.4) years; **(D)** IAA 28.8 (28.8-28.8) vs 27.4 (26.7-28-0). Log rank for a-c) *P*<0.001; **(D)** *P*<0.64.

**Figure 2:**
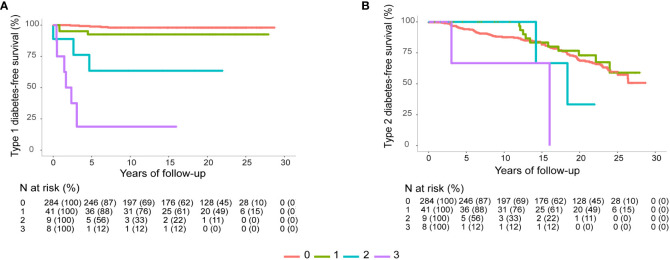
**(A)** Mean (95% CI) T1DM free survival time of women with no autoantibodies, 28.3 (27.9-28.7) years; one positive autoantibody, 26.8 (24.6-29.0) years; two positive autoantibodies, 19.2 (10.5-27.9) years; and three autoantibodies, 6.76 (-1.5-15.1) years. **(B)** Mean (95% CI) T2DM free survival time of women with no autoantibodies, 22.8 (21.7-23.8) years; one positive autoantibody, 24.2 (21.8-26.5) years; two positive autoantibodies, 20.5 (13.5-27.4) years; and three autoantibodies, 11.7 (4.8-18.6) years. Log rank *P*<0.0001 in both figures.

Positivity for ICA, GADA and/or IA-2A, but not for IAA, decreased T1DM-free survival time and time to diagnosis (**Figure 1**). All women who tested positive for three autoantibodies developed T1DM (**Figure 2A**). In contrast, T2DM-free survival rate and time to diagnosis were not significantly related to autoantibody positivity or negativity (**Figure 2B**).

Among women who later reported being diagnosed with T2DM (N=197), nine had two, and eight had three positive autoantibodies. Compared to the women reporting later T2DM but no autoantibodies detected, their first trimester BMI was lower (22.8 ± 5.1 vs 27.9 ± 6.2 kg/m^2^, p=0.306), however this, or the time to diagnosis (12.9 ± 6.8 vs 13.1 ± 7.2 years, p=0.408), did not reach statistical significance.

### 3.2 Prediction of diabetes progression after GDM

To analyse the influence of independent factors for T1DM or T2DM progression after GDM, two conditional logistic regression models were developed (**Table 2**). The highest risk of developing T1DM was associated with three positive autoantibodies, insulin treatment for GDM, and inversely associated with age under 30 years at the time of the GDM pregnancy. In terms of the individual autoantibodies, positivity for ICA was associated with the highest risk for T1DM progression, followed by GADA and IA-2A. This finding was supported by the ROC analyses, in which ICA positivity was the most predictive autoantibody regarding T1DM development (**Table 3**). The best predictive value was achieved by the combination of ICA and GADA positivity. Combination of ICA positivity and insulin treatment for GDM resulted in a highly sensitive, but less specific, prediction for T1DM. Despite some positive autoantibodies among those women who later developed T2DM, seropositivity was not significantly associated with the development of T2DM (**Table 2**).

**Table 2:** "Prediction of disease progression to T1 or T2 diabetes after GDM by independent factors using two logistic regression models.

	Type 1 diabetes	Type 2 diabetes
	OR (95% CI)	OR (95% CI)
Model 1		
No. of positive autoantibody types		
0	1.00	1.00
1	6.56 (1.52-28.34)	1.07 (0.55-2.08)
2	15.19 (3.54-65.15)	1.37 (0.33-5.59)
3	33.93 (8.95-128.66)	3.01 (0.72-12.49)
Age at time of GDM^*^		
≤ 30 years	1.00	1.00
> 30 years	0.24 (0.07-0.77)	1.65 (1.06-2.56)
Insulin for GDM		
No	1.00	1.00
Yes	13.44 (2.73-66.07)	3.74 (2.46-5.69)
Model 2		
ICA		
Negative	1.00	1.00
Positive	13.08 (3.60-47.56)	0.96 (0.45-2.02)
GADA		
Negative	1.00	1.00
Positive	5.21 (1.17-23.22)	1.38 (0.41-4.69)
IA-2A		
Negative	1.00	1.00
Positive	0.57 (0.12-2.76)	1.33 (0.40-4.41)
IAA		
Negative	NA**^§^**	1.00
Positive	NA**^§^**	2.36 (0.73-7.63)
Age at time of GDM		
≤ 30 years	1.00	1.00
> 30 years	0.47 (0.13-1.76)	1.91 (1.18-3.07)
Insulin for GDM		
No	1.00	1.00
Yes	28.37 (5.50-146.38)	3.73 (2.44-5.71)

*Age at the time of blood sampling during pregnancy.

**^§^** Not applicable due to small sample size.

**Table 3:** Autoantibody positivity or combination of autoantibodies and individual clinical factors in prediction of disease progression to T1DM by receiver operating characteristic (ROC) analyses; area under curve (AUC), sensitivity and specificity.

	Type 1 diabetes mellitus		
	AUC	Sensitivity (%)	Specificity (%)
Positivity of autoantibodies			
ICA	**0.77**	**64.7**	**90.1**
GADA	0.75	52.9	96.4
IA-2A	0.69	41.2	96.9
IAA	0.51		
Combinations of positive autoantibodies			
ICA + GADA	**0.82**	**70.6**	**88.0**
ICA + IA-2A	0.78	64.7	87.5
ICA + IAA	0.78	64.7	90.1
GADA + IA-2A	0.78	58.8	95.1
GADA + IAA	0.75	52.9	96.6
IA-2A + IAA	0.70	41.2	97.2
ICA + GADA + IA-2A	0.82	70.6	88.0
ICA + IAA + GADA	0.82	70.6	88.2
ICA + IAA + IA- 2A	0.79	64.7	87.8
GADA + IAA + IA-2A	0.78	58.8	95.3
Combinations of autoantibodies and insulin			
ICA + insulin	0.90	100.0	56.8
IAA + insulin	0.77	88.2	66.0
GADA + insulin	0.87	94.1	64.4
IA-2A + insulin	0.82	88.2	64.0
ICA + IAA + insulin	0.90	100.0	57.6
ICA + GADA + insulin	0.91	64.7	96.4
ICA + IA-2A + insulin	0.89	100.0	56.6
IAA + GADA + insulin	0.88	94.1	65.0
IAA + IA-2A + insulin	0.83	88.2	64.8
GADA + IA-2A + insulin	0.88	94.1	64.2
ICA + IAA + GADA + insulin	0.92	64.7	96.6
ICA + IAA + IA-2A + insulin	0.89	100.0	57.2
ICA + GADA + IA-2A + insulin	0.92	64.7	97.5
IAA + GADA + IA-2A + insulin	0.88	94.1	64.7

The most predictive values are marked in bold.

## 4 Discussion

This 23-year prospective cohort study showed that T1DM can be reliably predicted with ICA and GAD autoantibodies during pregnancy, and that progression to T1DM occurs during the first decade after GDM.

Development of T1DM results from the immune-mediated destruction of the pancreatic ß-cells. Presence of circulating autoantibodies produced by the B-lymphocytes is a well-characterized phenomenon, and they can be detected in the serum months to years before the onset of diabetes (26). Prevalence of autoantibodies in women with GDM has been previously described in several studies, including our own 6-year follow-up study of the same study population (15). Most studies have investigated the autoantibodies during pregnancy (7, 11, 13, 15, 17, 20, 21, 23–25), however, some studies investigated them after pregnancy (5, 14, 22) and one study both during and after the GDM pregnancy (12). Overall, GADA has been the most frequently assessed autoantibody, however, its prevalence (0-10.8%) and association to the progression to T1DM has varied considerably in different populations (5, 6, 8–10, 12, 13, 19, 21, 27–32), which probably at least partly reflects the differences of β-cell autoimmunity in various ethnic groups. Similarly, ICA prevalence has been variable (1-44%), but it may partly be due to technical issues regarding the standardization of the assays (33). The ICA assay applied in this study is highly sensitive (100%), adding to the reliability of our results. Here, IAA and IA-2A were not useful in predicting later T1DM after GDM, and this may reflect that they are more commonly found in young children and rarely in adults (34, 35). A novel β-cell autoantibody, ZnT8A, has been introduced since the initiation of our study, and initially, it was reported to have a prevalence of 4.8% in a GDM cohort (31). A subsequent study reported a lower prevalence 3.2%, while overall 6.8% of GDM women were autoantibody positive (32), and it seemed that ZnT8A provided no additional benefit above GADA positivity in terms of T1DM prediction.

In the present study, all women with three positive autoantibodies developed T1DM, which is in line with previous findings estimating that positivity for two autoantibodies increases the risk for developing T1DM to 63%, and up to 84%, when three autoantibodies are present (12). Here, the combination of ICA and GADA predicted T1DM with a 70.6% sensitivity and 88.0% specificity, and the prediction did not improve with an additional antibody analysed. Combination of any autoantibody and insulin treatment for GDM was very sensitive, but not a very specific predictor of T1DM progression, as it is also associated with later T2DM progression. We had as well eight women who had tested initially positive for three autoantibodies, yet self-reported being subsequently diagnosed with T2DM. Positivity for three autoantibodies strongly indicates that these patients do have autoimmune diabetes and not T2DM. In our view, these women most likely represent latent autoimmune diabetes in adults (LADA) that have been misdiagnosed in the primary care setting, where T2DM typically is treated in Finland. LADA may exhibit prolonged preservation of insulin secretion, and therefore a variable progression to insulin dependence, thus in the absence of antibody testing at the primary care setting, a misdiagnosis of T2DM is highly likely. The fact that they were slimmer supports this finding, although this difference did not reach statistical significance, most likely, due to a small sample size.

While results presented here and in previous studies seem conclusive that autoantibodies can effectively predict future T1DM, the main clinical question of whom to test for autoantibodies remains. In our population-based cohort, 5.7% of women with GDM developed T1DM (16), and therefore it is hardly clinically or economically sensible to consider autoantibody testing for all women with GDM, although that has been suggested (36). In this study, progression to T1DM was associated with GDM at the age below 30 years, insulin therapy and lower BMI, and these clinical factors would probably be most useful in the clinical decision making. In addition, presence of ketones and co-morbidity with other autoimmune diseases (such as hypothyroidism) have been proposed (37). In clinical practice, an atypical response to GDM treatment, e.g. no/little response to diet or metformin treatment, but strong response to insulin treatment indicates low insulin resistance, and is suggestive of insulin deficiency, thus justifying autoantibody testing.

Strengths of this study include a remarkably high participation rate (76%), and to our knowledge, the longest follow-up period to date. In addition, the GDM diagnosis was mainly (92.8%) based on OGTT, the gold standard for GDM diagnostics. We also investigated all four autoantibodies associated with diabetes progression instead of one or two typically seen in previous reports and were able to integrate significant clinical factors such as maternal age and BMI into the prediction models. However, self-reported data on disease progression is a weakness of this study, and a systematic OGTT on follow-up would have probably increased the prevalence of T2DM in both GDM and control cohorts. At the time of the study, a risk-based screening for GDM was used in Finland, which compared to the current nearly universal screening, may also underestimate the incidence of GDM. It is also noteworthy that the incidence of T1DM among young adults is higher in Finland than in other countries, which may diminish the generalisability of these results (38).

In conclusion, the presence of autoantibodies in first trimester samples of women with GDM predicts well later T1DM progression. The combination of ICA and GADA seems to be particularly sensitive and specific for this. Investigation of autoantibodies should be considered if GDM includes T1DM-like features, such as young age, low BMI or an atypical response to common GDM treatment.

## Data availability statement

The datasets presented in this article are not readily available because current GDPR legistlation does not allow transfer of data without the consent of the individuals who participated in the study. Requests to access the datasets should be directed to Juha S. Tapanainen, juha.tapanainen@helsinki.fi.

## Ethics statement

The studies involving humans were approved by Ethics Committee of the Northern Ostrobothnia Hospital District. The studies were conducted in accordance with the local legislation and institutional requirements. The participants provided their written informed consent to participate in this study.

## Author contributions

KL: Data curation, Investigation, Project administration, Visualization, Writing – original draft, Writing – review & editing, Supervision. AA: Data curation, Formal Analysis, Investigation, Writing – original draft, Writing – review & editing. JA: Data curation, Formal Analysis, Investigation, Methodology, Software, Validation, Writing – review & editing. JJ: Data curation, Formal Analysis, Investigation, Methodology, Software, Validation, Writing – review & editing, Visualization. IJ: Investigation, Writing – review & editing. MK: Investigation, Methodology, Resources, Writing – review & editing. JT: Conceptualization, Funding acquisition, Investigation, Project administration, Resources, Supervision, Writing – review & editing.
